# Hck promotes IL-1β-induced extracellular matrix degradation, inflammation, and apoptosis in osteoarthritis via activation of the JAK-STAT3 signaling pathway

**DOI:** 10.1186/s42358-024-00427-2

**Published:** 2024-12-18

**Authors:** Zhenzhong Yan, Lin Ji

**Affiliations:** 1 Department of Orthopedics, Changzhou No. 7 People’s Hospital, No. 288 Yanling East Road, Economic Development Zone, Changzhou, Jiangsu 213100 China; 2 Department of Orthopedics, Changzhou Geriatric Hospital Affiliated to Soochow University, No. 288 Yanling East Road, Economic Development Zone, Changzhou, Jiangsu 213100 China

**Keywords:** Osteoarthritis, Hck, Chondrocytes, Apoptosis extracellular matrix, JAK-STAT3

## Abstract

We investigated role of haematopoietic cell kinase (Hck) in osteoarthritis (OA) and to explore the underlying mechanisms driving its effects. An OA animal model was established and after OA induction, rats received intra-articular injections of lentivirus twice a week for four weeks. Rats were divided into four groups: control (healthy rats without OA), OA model (rats with induced OA), OA + Len-si-NC (OA rats treated with a non-targeting control lentivirus), and OA + Len-si-Hck (OA rats treated with lentivirus targeting Hck). Blood samples were collected, and serum cytokine levels were measured using ELISA. Afterward, the rats were sacrificed for histological analysis and TUNEL assay. In vitro, IL-1β-treated human chondrocytes were transfected with Hck, and the effects on cell viability, apoptosis, ECM degradation, and JAK-STAT3 signaling were assessed. Colivelin, a JAK-STAT3 agonist, was used to confirm the pathway’s involvement. Results indicated increased Hck expression in the cartilage tissues of OA rats and in IL-1β-stimulated chondrocytes. Silencing Hck i*n vivo* reduced IL-6 and TNF-α levels, apoptosis, and preserved cartilage structure. In vitro, Hck knockdown in IL-1β-treated chondrocytes resulted in enhanced cell viability, reduced apoptosis, and decreased ECM degradation. Notably, the expression of MMP3 and MMP13 was significantly lowered, while collagen II and aggrecan levels were restored. Additionally, Hck knockdown inhibited JAK-STAT3 activation, which was evident from reduced levels of phosphorylated JAK1 and STAT3. The addition of colivelin reversed these effects, confirming that Hck mediates its effects through the JAK-STAT3 pathway. Overall, our findings indicate that Hck is critical in OA progression by promoting inflammation, apoptosis, and ECM degradation through the JAK-STAT3 signaling pathway activation.

**Supplementary Information:**

The online version contains supplementary material available at 10.1186/s42358-024-00427-2.

## Introduction

Osteoarthritis (OA) is a degenerative joint disease that significantly lowers quality of life. It is characterized by cartilage loss, ongoing joint discomfort, and decreased mobility [37]. The pathogenesis of OA includes aging, mechanical stress, obesity, metabolic disorders, and chronic inflammation. These factors collectively contribute to alterations in the joint structure, including the reduction of synovial fluid, changes in cartilage composition, and the formation of osteophytes [29]. Recent studies have emphasized that inflammation and cellular signaling pathways are critical in OA progression [7, 12, 37]. Adipose tissue secretes various adipokines and inflammatory cytokines which exacerbate cartilage degradation and chondrocyte apoptosis [20, 31]. The extracellular matrix (ECM) of cartilage, primarily composed of collagen II and aggrecan, undergoes degradation due to elevated activity of matrix metalloproteinases (MMPs), further advancing OA [42]. Current therapeutic approaches for OA focus on symptom management through analgesics, anti-inflammatory agents, and physical therapy, with surgical interventions reserved as a last resort [19, 32]. However, the exact molecular pathway and mechanism driving OA progression remain unclear, necessitating further research to identify novel therapeutic targets.

Src family kinases (SFKs) have emerged as critical regulators of cellular signaling, implicated in various diseases, including OA [1, 5, 15]. Haematopoietic cell kinase (Hck) is a member of the SFKs and found in myeloid and B-lymphocyte cells [3]. Hck has been linked to several pathological conditions, particularly hematological malignancies and solid tumors, through its interaction with receptor tyrosine kinases and activation of signaling pathways like JAK/STAT, MEK/ERK, and PI3K/AKT [22, 40]. The JAK-STAT3 signaling system, which regulates cell proliferation, differentiation, and death, has been linked to OA pathogenesis [18, 38]. Although the role of Hck in solid tumors is well-documented, its involvement in OA, particularly through the JAK-STAT3 pathway, remains underexplored. We have undertaken this study to examine role of Hck in OA progression via JAK-STAT3 signaling pathway. By elucidating the molecular mechanisms through which Hck contributes to OA, this research aims to identify Hck as a critical target for treating OA.

## Materials and methods

### OA model

Twenty adult SD rats (10 weeks old) from Vital River Technology Beijing, China were purchased and divided into control, OA model, OA + Len-si-NC, and OA + Len-si-Hck (*n* = 5/group). The OA model was developed through medial collateral ligament transection and medial meniscus instability in the right knee joint. Rats were anesthetized using 0.5% pentobarbital sodium (i.p, 100 mg/kg) to induce anesthesia. Postoperative care included monitoring for signs of pain and infection, with appropriate interventions as needed. Lentiviral vectors expressing Hck shRNA (Len-si-Hck) and negative control shRNA (Len-si-NC) were constructed using the pLenti-EF1a-EGFP-F2A-Puro vector backbone (Biovector, China). The specific sequence targeting Hck was 5’-GACAGAUCUUGCUCAACUU-3’. One week after OA induction, rats in the respective treatment groups received intra-articular injections of the lentivirus (1 × 10⁹ PFU in 20 µl PBS) twice a week for four weeks under aseptic conditions. Two months after the initial surgery, blood samples were taken from orbital venous plexus to examine levels of inflammatory cytokines using ELISA kits (R&D Systems, China). Subsequently, rats were sacrificed by CO₂ inhalation, and articular cartilage tissues from the knee joints were harvested for histological and molecular analyses. The study received approval from the university’s ethics committee.

### Hematoxylin and eosin (H&E) staining

Articular cartilage tissues were fixed in 10% formalin and decalcified in 10% EDTA for 14 days. The tissues were embedded in paraffin, sectioned into thin slices, dewaxed and rehydrated. Finally, the sections were stained with H and E solution (Beyotime, China), dehydrated through a series of alcohols, and sealed with mounting media. Cartilage morphology was examined using a Nikon microscope (Japan). Two independent and experienced researchers, who were blinded to the study, assessed cartilage degenerative changes using the Osteoarthritis Research Society International (OARSI) Scoring System as described previously [27].

### TUNEL

To assess apoptosis within the cartilage, we employed the TUNEL assay using a kit from Beyotime (USA). For each rat, ten random areas were selected from the cartilage tissue sections for analysis. The TUNEL-positive cells were quantified under a Nikon microscope.

### Chondrocyte culture, treatment and transfection

Human chondrocytes (C28/I2 cells) from Sigma-Aldrich company were cultured in 10% DMEM. The chondrocytes were treated with interleukin-1β (IL-1β), at 10 ng/ml (Sigma-Aldrich, USA) for 24 h to mimic an OA cell model. Once cells reached 70% confluence, they were divided into the following groups: Control (untreated), IL-1β, IL-1β + si-NC, IL-1β + si-Hck#2, and IL-1β + si-Hck#2 + colivelin. The IL-1β + si-Hck#2 + colivelin group was treated with 0.5 µM colivelin (MedChemExpress, USA) for 6 h post transfection to investigate whether JAK-STAT3 signaling mediated the effects of Hck. Notably, colivelin treatment was specifically applied only to the IL-1β + si-Hck#2 group to assess whether it could counteract the effects of Hck silencing. Three small interfering RNAs (siRNAs, GenPharma (Shanghai, China) targeting Hck were designed with the following sequences: si-Hck#1: 5’-GCUAUGACCUAUCGGAUUU-3’, si-Hck#2: 5’-CGUAGAUCAUACGGAUUAU-3’, si-Hck#3: 5’-AUCGUAUGCCGAUUACGAA-3’. A negative control siRNA (si-NC) with the following sequence was also used: si-NC: 5’-UUCUCCGAACGUGUCACGU-3’. Cell transfection was done with Lipofectamine 3000 with the transfection lasting for 48 h.

### ELISA

Human chondrocytes seeded in a 24-well plate were treated under the experimental conditions. The cell culture medium was carefully collected to avoid disturbing the cells. The concentrations of TNF-α and IL-6 in the rats supernatant were measured using ELISA kits. The absorbance was read at specified wavelength, and the cytokine levels were calculated using standard curves.

### CCK-8

Human chondrocytes were maintained in 96-well plate and treated with 100 µl of CCK-8 reagent (Sigma-Aldrich, USA) to each well. The plate was placed in a humidified chamber with 5% CO2 and incubated for two hours at 37 °C. Cell viability was assessed following incubation by measuring the absorbance at 450 nm using a microplate reader (Thermo Fisher Scientific, USA).

### Flow cytometry

Human chondrocytes 1 × 10⁵ cells/sample) were trypsinized, mixed in binding buffer and then stained with PI/FITC-Annexin V (BD Biosciences, USA) for 15 min. After staining, the samples were analyzed by flow cytometry (BD Biosciences, USA). Data were recorded and evaluated to investigate percentage of apoptotic cells in each group.

### RT-qPCR

Both tissues and cells were lysed using TRIzol reagent, after which cDNA was synthesized with help of PrimeScript RT Master Mix (Takara, Japan). QRT-qPCR was performed using miScript SYBR^®^-Green PCR kit (Thermo Fisher Scientific, USA). Gene expression levels were analyzed using the 2^−ΔΔCt^ method. Sequence used for Hck was Fwd: 5’-GACAGAUCUUGCUCAACUU-3’ and Rev: 5’-AAGGCUAUCAGCUCGUUAU-3’.

### Western blot

Proteins were extracted using RIPA buffer (Beyotime, USA) and quantified with a BCA protein assay kit (Beyotime, USA). After being separated by 10% SDS-PAGE, the extracted proteins were put onto a PVDF membrane which was then blocked with 5% milk solution to prevent nonspecific binding. Following blocking, Primary and secondary antibodies were first incubated on the membrane. With the use of an ECL detection kit (Beyotime, USA), protein bands were visible. Table 1 Supplementary List contains the list of antibodies.

### Statistical analysis

To ensure reproducibility, each experiment was run at least three times. To do statistical analysis, GraphPad Prism 6.0 was used. The mean ± standard deviation (SDwas used to display the data. Tukey’s multiple comparison test was conducted after the Student’s t-test or one-way analysis of variance (ANOVA) was used to evaluate the differences between the groups.

## Results

### Hck knockdown inhibits inflammation and chondrocyte apoptosis in OA rats

**Fig. 1:**
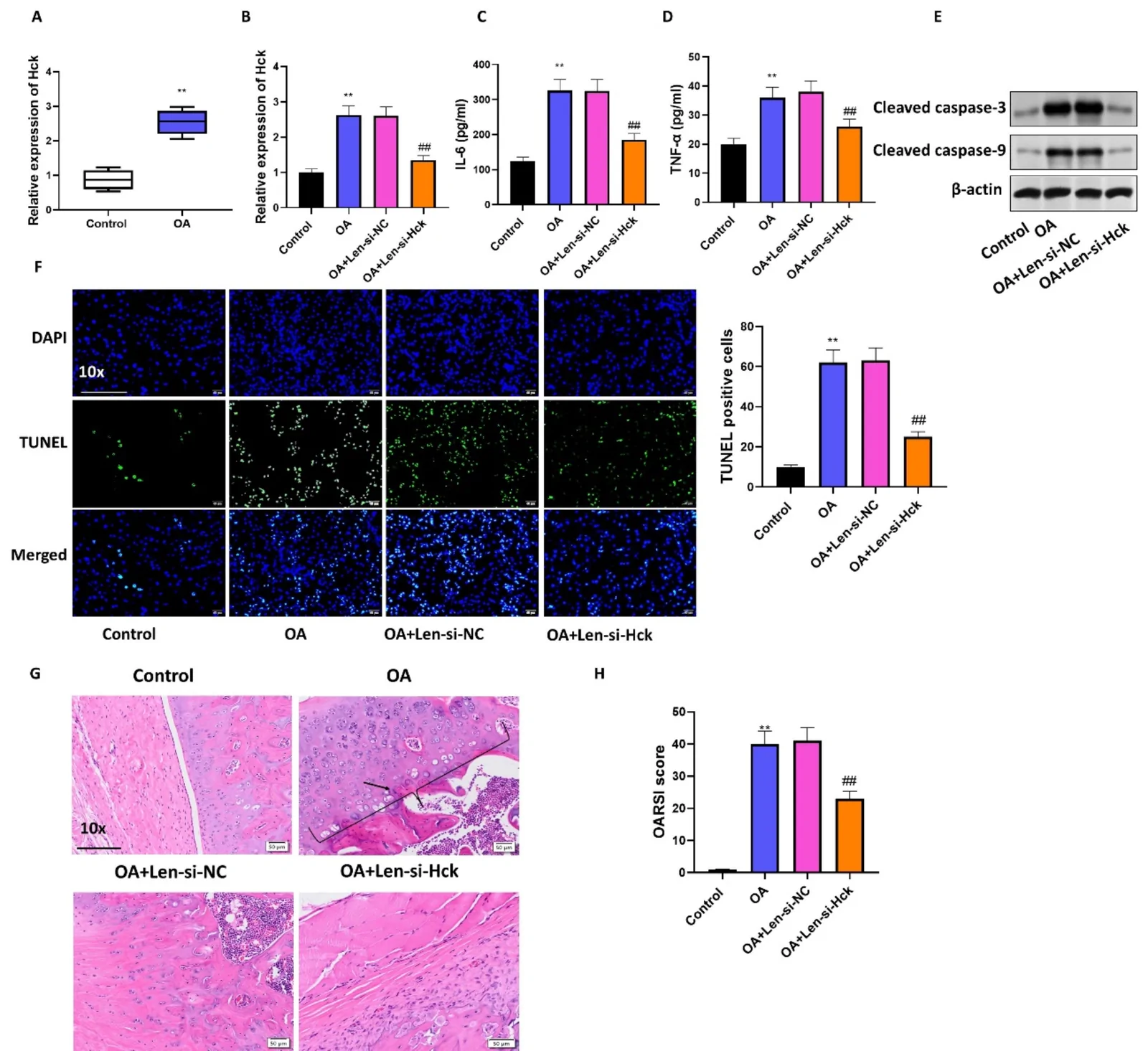
Hck Knockdown Reduces Inflammation and Apoptosis in OA Rats. **A** Hck levels in OA tissues and normal tissues of rats were investigated using RT-qPCR. **B** After treatment with Len-si-Hck, Hck expression in OA rats was detected by RT-qPCR. **C**-**D** ELISA was used to measure levels of IL-6 and TNF-α in the rats’ serum in OA rats or Len-si-Hck treated OA rats. **E** Western blot examined the levels of apoptotic proteins in OA rats or Len-si-Hck treated OA rats. **F** The TUNEL assay assessed the apoptosis in chondrocytes in OA rats or Len-si-Hck treated OA rats. **G** H&E staining assessed the pathological changes of the articular cartilage tissues in OA rats or Len-si-Hck treated OA rats. Arrow and the bracket represented damaged region of the articular cartilage tissues. **H** OARIS scores for articular cartilage in OA rats or Len-si-Hck treated OA rats. **P < 0.01, compared to control group; ##*P* < 0.01, compared to OA group

We analyzed Hck levels in the cartilage of rats with OA using RT-qPCR. Our findings indicate that relative to control group, Hck was higher in the OA group (Fig. 1A), suggesting the involvement of Hck in OA. To test this, we used lentivirus to reduce Hck expression. As shown in Fig. 1B, the lentivirus effectively lowered Hck levels in the rats. Following this intervention, we observed that relative to control group, the levels of IL-6 and TNF-α were elevated in the OA rats, but after Hck was silenced, the levels of IL-6 and TNF-α were reduced in the OA rats (Fig. 1C-D). In addition, relative to control group, the levels of cleaved caspase-3 and caspase-9 were higher in the OA group, but their levels were reduced after Hck knockdown (Fig. 1E). TUNEL staining further supported the findings, showing an increase in TUNEL-positive cells in the OA group compared to the control group, while the number of TUNEL-positive cells was reduced in the OA + Len-si-Hck group (Fig. 1F). Histological examination indicated that control group had smooth cartilage with well-organized chondrocytes. In contrast, the OA group displayed thinning cartilage with fissures and disorganized chondrocytes. However, the OA + Len-si-Hck group showed a relatively smooth cartilage surface with more regular chondrocyte alignment, although the surface was slightly rougher (Fig. 1G). The OARSI scores in the OA group were significantly higher than those in the control group, while the OA + Len-si-Hck group had notably lower OARSI scores compared to the OA group (Fig. 1H).

### Hck silencing reduces IL-1β-induced ECM degradation, inflammation, and apoptosis

**Fig. 2:**
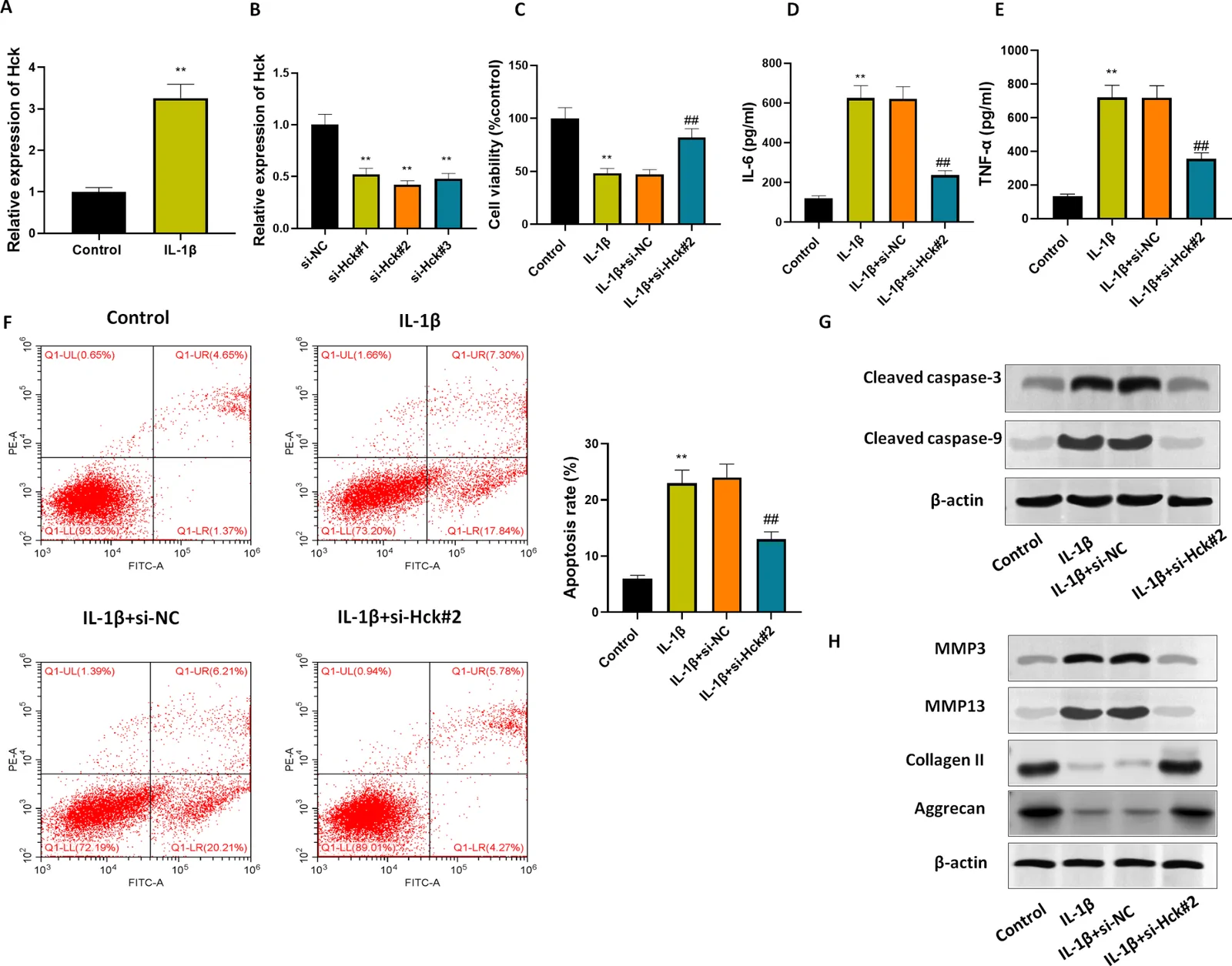
Hck Knockdown Reduces ECM Degradation, Inflammation, and Apoptosis in Chondrocytes. **A** Hck level in chondrocytes treated with IL-1β, measured by RT-qPCR. **B** RT-qPCR confirmed the effectiveness of siRNA transfection in reducing Hck levels. **C** Cell viability was evaluated with CCK-8 test in IL-1β-treated chondrocytes following Hck knockdown. **D**-**E** ELISA measured IL-6 and TNF-α levels in IL-1β-treated chondrocytes following Hck knockdown. **F** Apoptosis in IL-1β-treated chondrocytes was assessed by flow cytometry analysis after Hck was silenced. **G**-**H** The levels of proteins related to apoptosis and ECM in IL-1β-treated chondrocytes following Hck knockdown was measured by western blot. *P* < 0.01 in contrast to the control group; ##*P* < 0.01 in contrast to the group receiving IL-1β

We next explored how silencing Hck affects human chondrocytes when exposed to IL-1β. Initially, we found that IL-1β treatment in chondrocytes led to an increase in Hck expression (Fig. 2A). To dive deeper, we used three different siRNAs to knock down Hck, and RT-qPCR confirmed that all three reduced Hck levels, with si-Hck#2 being the most effective (Fig. 2B). Consequently, si-Hck#2 was used in subsequent experiments. Compared to the control group, IL-1β typically reduced chondrocyte proliferation, but when Hck was silenced with si-Hck#2, the cell viability was improved (Fig. 2C). Compared to the control group, treatment with IL-1β increased the production of inflammatory cytokines (IL-6 and TNF-α), but silencing Hck reduced their levels (Fig. 2D and E). Additionally, compared to the control group, IL-1β heightened the rate of apoptosis in chondrocytes, but this phenomenon was reduced after Hck silencing (Fig. 2F). Additionally, compared to the control group, IL-1β elevated cleaved caspase-3 and caspase-9 levels, but again, their levels were reduced when Hck was silenced (Fig. 2G). Finally, we investigated expression of ECM degradation proteins. Compared to the control group, IL-1β increased the levels of MMP3 and MMP13, while decreasing the levels of Collagen II and Aggrecan. However, silencing Hck reversed these effects, reducing MMP3 and MMP13 and restoring Collagen II and Aggrecan levels in IL-1β-treated human chondrocytes (Fig. 2H).

### Hck drives IL-1β-Induced ECM degradation, inflammation, and apoptosis in chondrocytes by activating JAK-STAT3 signaling

**Fig. 3:**
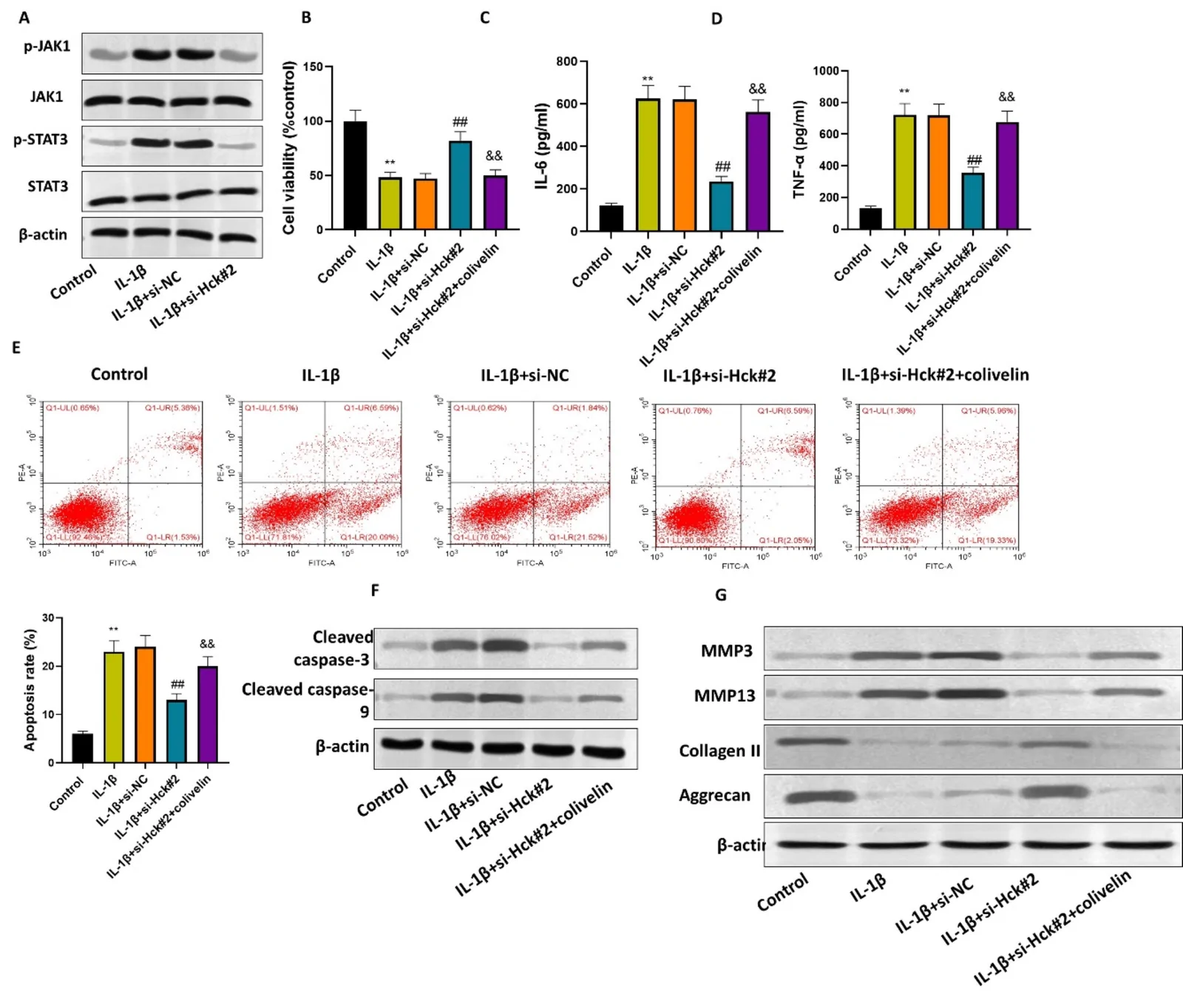
Hck enhances IL-1β-induced ECM degradation, inflammation, and apoptosis in chondrocytes via JAK-STAT3 signaling pathway. **A** Western blot analysis of JAK-STAT3 signaling proteins in IL-1β-stimulated chondrocytes following Hck knockdown and colivelin treatment. **B** CCK-8 assay measuring cell viability in IL-1β-stimulated chondrocytes following Hck knockdown and colivelin treatment. **C**-**D** ELISA examined the levels of inflammatory markers in IL-1β-stimulated chondrocytes following Hck knockdown and colivelin treatment. **E** Cell apoptosis in IL-1β-stimulated chondrocytes following Hck knockdown and colivelin treatment was measured by flow cytometry. **F**-**G** Western blot analyzed the levels of apoptosis and ECM-related proteins in IL-1β-stimulated chondrocytes following Hck knockdown and colivelin treatment. *P* < 0.01, vs. control group. ##*P* < 0.01, vs. IL-1β group. &&*P* < 0.01, vs. IL-1β-si-Hck#2 group

Previous studies have shown that the JAK-STAT3 signaling pathway influences OA progression [43] and that Hck can activate JAK-STAT3 in various diseases [17]. Thus, we believed that Hck might contribute to OA progression via JAK-STAT3 pathway. Western blot analysis revealed upregulated levels of phosphorylated JAK and STAT3 (p-JAK and p-STAT3) by IL-1β which were significantly reduced after transfection with si-Hck#2 (Fig. 3A). To further investigate whether Hck’s effects on OA are mediated through JAK-STAT3, we treated the cells with colivelin, a JAK-STAT3 pathway agonist. The results showed that colivelin counteracted the increased cell viability in HCK-silenced chondrocytes in the presence of IL-1β (Fig. 3B). Additionally, in the presence of IL-1β, the reduced IL-6 and TNF-α levels in si-Hck#2-transfected chondrocytes were reversed following colivelin treatment (Fig. 3C-D). Furthermore, in the presence of IL-1β, the decreased apoptosis as well as the reduced expression of cleaved caspase-3/9 caused by Hck knockdown were also reversed after colivelin addition (Fig. 3E and F). Similarly, in the presence of IL-1β, the reduced levels of MMP3 and MMP13 along with the increased levels of Collagen II and Aggrecan in si-Hck#2-transfected chondrocytes were reversed with colivelin treatment (Fig. 3G).

## Discussion

Hck plays a pivotal role in cellular signaling pathways [35] and its overactivation has been associated with pathological conditions, including hematological malignancies and solid tumors [4, 16, 25]. HCK drives the release of pro-inflammatory signals and growth factors from myeloid cells, shifting macrophages towards a phenotype that aids in tissue repair and supports tumor growth [23]. Recent research has demonstrated the therapeutic potential of targeting HCK inhibition in various cancers. A novel compound, iHCK-37, was found to exhibit anti-neoplastic activity in leukemia cells, suggesting its potential as a therapeutic agent for acute myeloid leukemia [28]. Additionally, inhibition of HCK activity has been shown to suppress the progression of colon cancer mediated by myeloid cells [24]. Notably, targeting HCK not only suppresses tumor growth but also enhances the efficacy of immunotherapy by both activating immune cells and reducing the immunosuppressive tumor microenvironment [26]. These findings highlight the benefits of HCK inhibition in cancer treatment. Although previous research has explored Hck expression in OA [9], the exact role and underlying mechanisms of Hck in OA pathogenesis have remained largely unexplored. We present the first study that Hck activates the JAK-STAT3 signaling pathway in chondrocytes, thereby inducing inflammation and apoptosis and OA development.

Chondrocyte dysfunction is a key factor in cartilage damage during OA [36]. The caspase family, particularly cleaved caspase-3 and caspase-9, plays a crucial role in apoptosis. We observed that IL-1β induced apoptosis in chondrocytes by increasing these cleaved caspases, consistent with previous studies [33]. In addition to apoptosis, inflammation is a hallmark of OA, with IL-1β elevating levels of pro-inflammatory cytokines in chondrocytes [21, 34]. Furthermore, IL-1β-induced ECM degradation is a major pathological feature of OA, characterized by breakdown of essential ECM components like collagen II and aggrecan, mediated by matrix metalloproteinases such as MMP3 and MMP13 [10]. Importantly, we demonstrated that Hck is upregulated in the cartilage tissues of OA rats and in IL-1β-stimulated chondrocytes (C28/I2 cells). Hck silencing not only reduced inflammatory response but also prevented IL-1β-induced ECM degradation, while promoting cell proliferation in chondrocytes. These results suggest a strong link between Hck and OA development, which is consistent with earlier literature on the involvement of SFKs in OA [2]. HCK has also been implicated in promoting renal inflammation and fibrosis, suggesting its broader role in inflammatory processes [3]. Inhibition of HCK through compounds like A-419,259 has shown promise as an anti-osteoclastogenic agent, offering potential for treating inflammatory bone destruction [14]. These findings reinforce the therapeutic potential of HCK inhibitors in various inflammatory conditions.

To further investigate the mechanisms by which Hck influences OA, we focused on the JAK-STAT3 signaling pathway, which helps in cell growth, proliferation, and differentiation [13, 39]. This pathway has been linked to the inflammatory mechanisms that underlie OA [6, 11]. Our results confirmed that IL-1β increased phosphorylated levels of JAK1 and STAT3, which were significantly reduced following Hck inhibition. This suggests that Hck promotes OA progression by activating the JAK-STAT3 pathway. Moreover, treatment with colivelin [8], a JAK-STAT3 pathway agonist, reversed the protective effects of Hck inhibition on IL-1β-stimulated chondrocyte apoptosis, inflammation, and ECM degradation, further supporting the involvement of this pathway in Hck-mediated OA pathogenesis. Consistent with these findings, blocking the JAK-STAT3 signaling pathway could be beneficial for rheumatoid arthritis treatment [30]. Activation of JAK-STAT3 has also been shown to drive inflammation in chondrocytes during OA [11]. Hck is closely associated with the pathogenesis of various human diseases through its interaction with the JAK/STAT pathway [41].

Our study has some limitations. First, only IL-1β was used to model OA in vitro, leaving uncertainty as to whether other models would yield the same outcomes. Second, we did not inhibit the STAT3 pathway in our in vivo mouse models to test whether blocking this signaling could reverse the damage. Lastly, the study did not include human synovial OA tissue, making it unclear if these findings would apply to human models. To fully understand role of Hck in OA, further detailed experiments are necessary.

In summary, our research shows that Hck is a major driver of the advancement of OA via stimulating the JAK-STAT3 signaling pathway. These results imply that focusing on Hck may be a viable approach to creating novel OA therapies.

## Electronic supplementary material

Below is the link to the electronic supplementary material.

Supplementary Material 1

Supplementary Material 2

## Data Availability

No datasets were generated or analysed during the current study.
