# Supplementary material for: Hck promotes IL-1β-induced extracellular matrix degradation, inflammation, and apoptosis in osteoarthritis via activation of the JAK-STAT3 signaling pathway
Source: Adv Rheumatol. 2024 Dec 18;64:88. doi: 10.1186/s42358-024-00427-2 (PMC13488854; doi:10.1186/s42358-024-00427-2)
Supplement: Supplementary file 1 [file 42358_2024_427_MOESM2_ESM.docx]

Figure 1E

2G

2H

**Aggrecan**

**Collagen**

**MMP3**

**Figure 3A**

**JAK**

**p-JAK**

**p-STAT**

**STAT**

**Figure 3F**

**Fig 3G**

**Aggrecan**

**Collagen**

**MMP3**

**MMP13**
